# Cholecystocolonic Fistula With Acute Cholangitis: A Case Report

**DOI:** 10.7759/cureus.115423

**Published:** 2026-08-30

**Authors:** Leon Yeung, Amos Nepacina Liew, Niyaz Naqash

**Affiliations:** 1 Department of Upper Gastrointestinal and Hepatobiliary Surgery, Monash Health, Melbourne, AUS

**Keywords:** acute cholangitis, cholecystocolonic fistula (ccf), cholecystoenteric fistula, laparoscopic cholecystectomy (lc), s: choledocholithiasis

## Abstract

Cholecystocolonic fistula (CCF) is a rare type of cholecystoenteric fistula, which are sometimes diagnosed incidentally in patients undergoing cholecystectomy. They are usually a complication of chronic cholecystitis, often secondary to gallstones, and they are difficult to diagnose due to their rarity and vague clinical presentation. If untreated, they can lead to complications such as acute cholangitis, biliary peritonitis, and biliary cirrhosis. This highlights the importance of early recognition of CCF and timely treatment. While the generally accepted management is surgical, there are no consensus guidelines available. We present a case of a 79-year-old with a CCF, diagnosed preoperatively in the setting of workup for acute cholangitis and choledocholithiasis. She presented with four days of right-sided abdominal pain and one day of vomiting and new jaundice. She was initially hypotensive with elevated inflammatory markers, hyperbilirubinaemia, and deranged liver function tests. Contrast-enhanced CT revealed an 11 mm distal common bile duct calculus with hepatic biliary dilatation, gas in the gallbladder lumen, and a large gallstone in the hepatic flexure, which were suggestive of an underlying CCF. She underwent a laparoscopic converted to open cholecystectomy with transcystic bile duct exploration and choledochotomy, as well as right hemicolectomy due to extensive oedema and inflammation surrounding the CCF. This was later followed by endoscopic retrograde cholangiopancreatography (ERCP) and biliary sphincterotomy. Her CCF and acute cholangitis were thus successfully treated with surgical intervention, and she recovered well. The rare occurrence of CCF highlights the importance of having a high index of clinical suspicion preoperatively as well as maintaining surgical flexibility if it is encountered during an operation. Early recognition and surgical intervention of CCF are important to prevent life-threatening complications.

## Introduction

A cholecystoenteric fistula (CEF) is a rare and abnormal connection between the gallbladder and the gastrointestinal tract. Cholecystocolonic fistula (CCF) is a rare type of CEF between the gallbladder and the colon, usually developing in patients with chronic cholecystitis, often secondary to cholelithiasis [1-3]. CCFs are difficult to diagnose due to their rare occurrence and vague clinical presentation and are often only found incidentally intraoperatively during cholecystectomy. If untreated, they can lead to life-threatening complications, including acute cholangitis, biliary peritonitis, and biliary cirrhosis [4], which highlights the importance of early recognition and appropriate treatment of CCF by surgeons and clinicians. However, while the generally accepted management is surgical, there are no consensus guidelines available. Therefore, we present a case of CCF treated successfully with surgical management and aim to summarise the currently available literature on CCF diagnosis and treatment.

## Case presentation

This case report and review of the literature was conducted according to the SCARE (Surgical CAse REport) consensus statement and CARE (CAse REport) guidelines. The patient provided informed consent for their de-identified medical information to be used. Herein, we report the case of a 79-year-old female patient who underwent a laparoscopic converted to open cholecystectomy with transcystic bile duct exploration, choledochotomy, and right hemicolectomy for a CCF.

History and examination

A 79-year-old female patient with no significant past medical history presented to the emergency department at our tertiary centre with four days of intermittent right-sided upper and lower abdominal pain, associated with a one-day history of vomiting and new jaundice. She denied any changes in bowel motions or urinary symptoms. She had a 30 pack-year smoking history and social alcohol intake. On examination, she was initially hypotensive with a blood pressure of 90/72 mmHg, which was fluid responsive. Her other observations were within normal limits, and she was afebrile. She had a soft abdomen with some tenderness in the right upper quadrant.

Investigations

Pathology results showed leukocytosis with a white cell count of 13.7 x 10^9^/L, neutrophil count of 13.2 × 10^9^/L, and C-reactive protein level of 275 mg/L. She had a pH of 7.45, lactate of 3.0, and haemoglobin of 121 g/L. Her platelet count was 95 x 10^9^/L, and she had mild hypokalaemia at 3.3 mmol/L. She also had deranged liver function tests, with bilirubin of 131 mcmol/L, alkaline phosphatase of 270 U/L, gamma-glutamyl transferase of 313 U/L, and alanine aminotransferase of 85 U/L. Her lipase level was 8 U/L, estimated glomerular filtration rate was 74 mL/min, and she had an albumin of 26 g/L. Based on the pathology results, she was commenced on broad spectrum empiric antibiotics for cholangitis, which were later switched to targeted therapy for confirmed *Escherichia coli* and *Streptococcus anginosus* bacteraemia.

Her initial CT abdomen and pelvis with contrast revealed an 11 mm distal common bile duct calculus with biliary duct dilatation (Figure 1), intraluminal gas within the gallbladder and pneumobilia (Figure 2), as well as a calcified object (likely a large gallstone) in the hepatic flexure (Figure 3). The impression was that this was a CCF with concurrent cholangitis and choledocholithiasis. 

**Figure 1:**
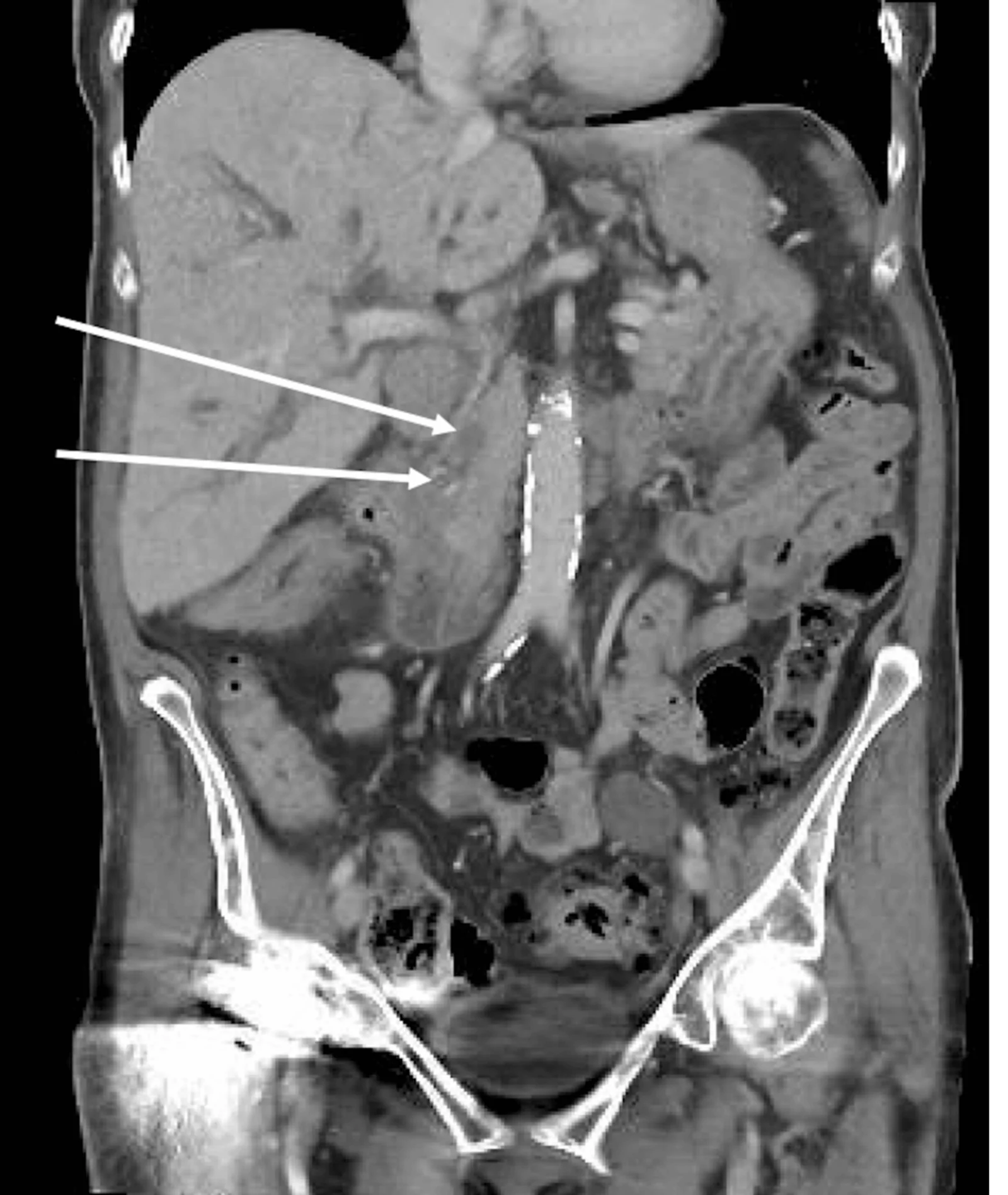
Coronal CT of the abdomen and pelvis demonstrating biliary duct dilatation (superior arrow) and a distal common bile duct calculus (inferior arrow).

**Figure 2:**
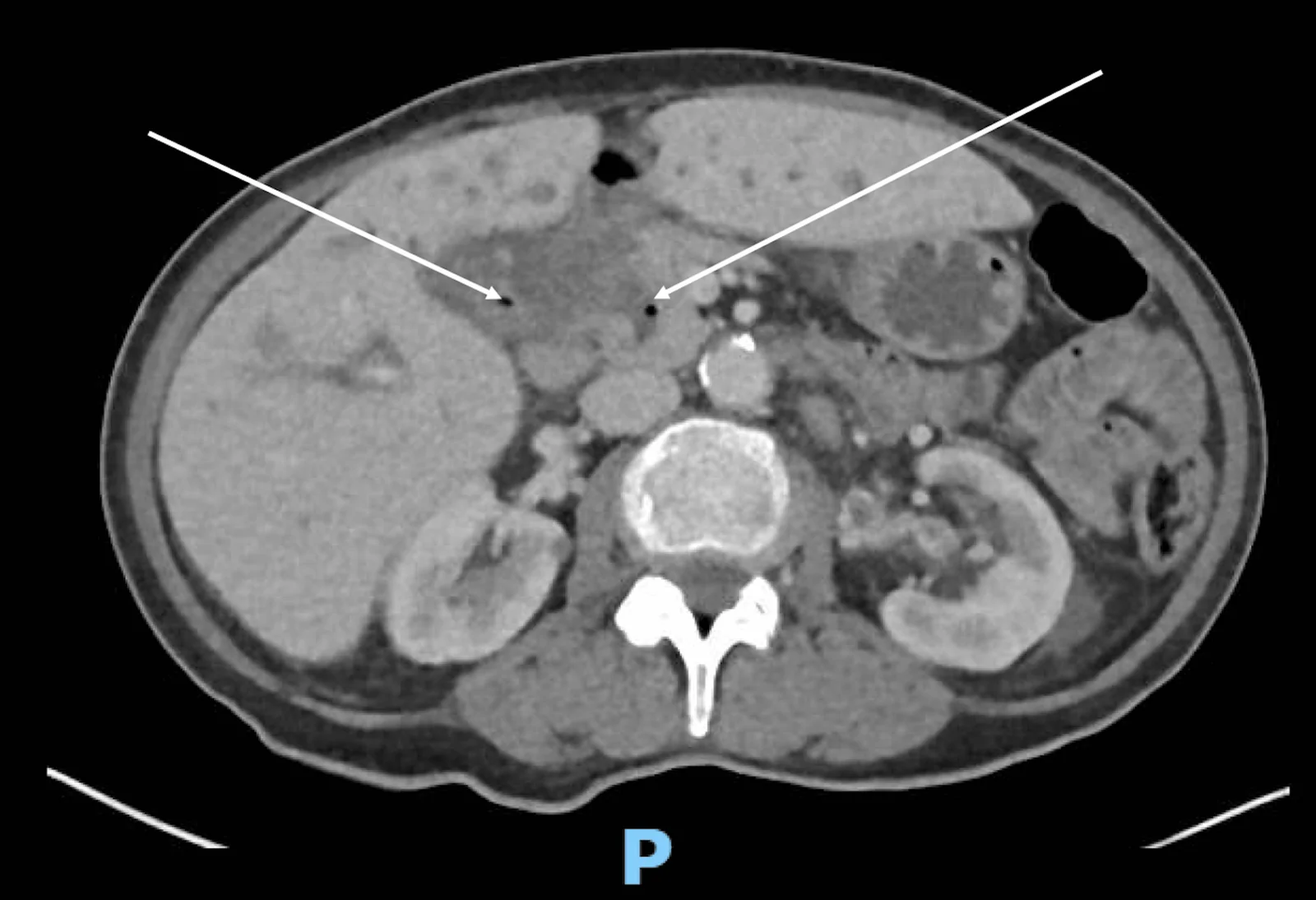
Axial CT of the abdomen and pelvis demonstrating intraluminal gas in the gallbladder (left arrow) and pneumobilia within a dilated common bile duct (right arrow).

**Figure 3:**
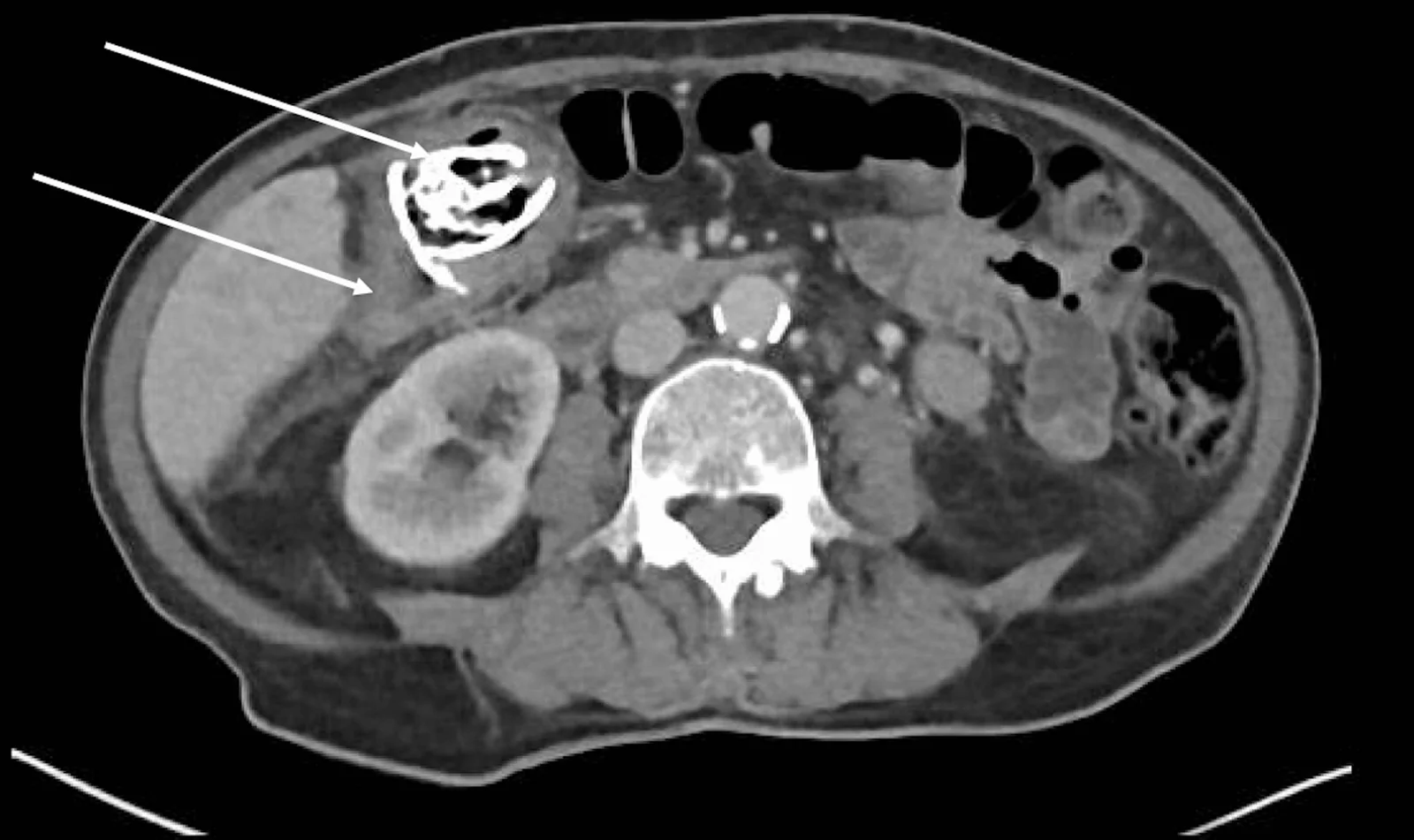
Axial CT of the abdomen and pelvis demonstrating a large calcified foreign object (superior arrow), likely a gallstone, within the hepatic flexure with associated colonic wall thickening (inferior arrow) and pericolonic stranding.

Management

She proceeded to undergo a laparoscopic converted to open cholecystectomy with transcystic bile duct exploration, choledochotomy, and right hemicolectomy. Intraoperative cholangiogram confirmed the presence of at least two large stones in the common bile duct (Figure 4). A flexible cystoscope was used for transcystic bile duct exploration, which was able to extract some of the stones, but one large stone in the distal bile duct could not be removed. There was confirmed cholangitis with purulent bile seen in the biliary tree. The decision was then made to convert to open via a transverse right subcostal incision, and the remaining gallstone was extracted from the distal common bile duct via longitudinal anterior choledochotomy. Right hemicolectomy was then performed due to extensive surrounding oedema and inflammation around the CCF, including removal of the large intraluminal colonic gallstone. Subsequent intraoperative gastroscopy confirmed no duodenal perforation or fistula involvement. The cystoscope was used once more to confirm clearance of the bile duct prior to closure of the choledochotomy.

**Figure 4:**
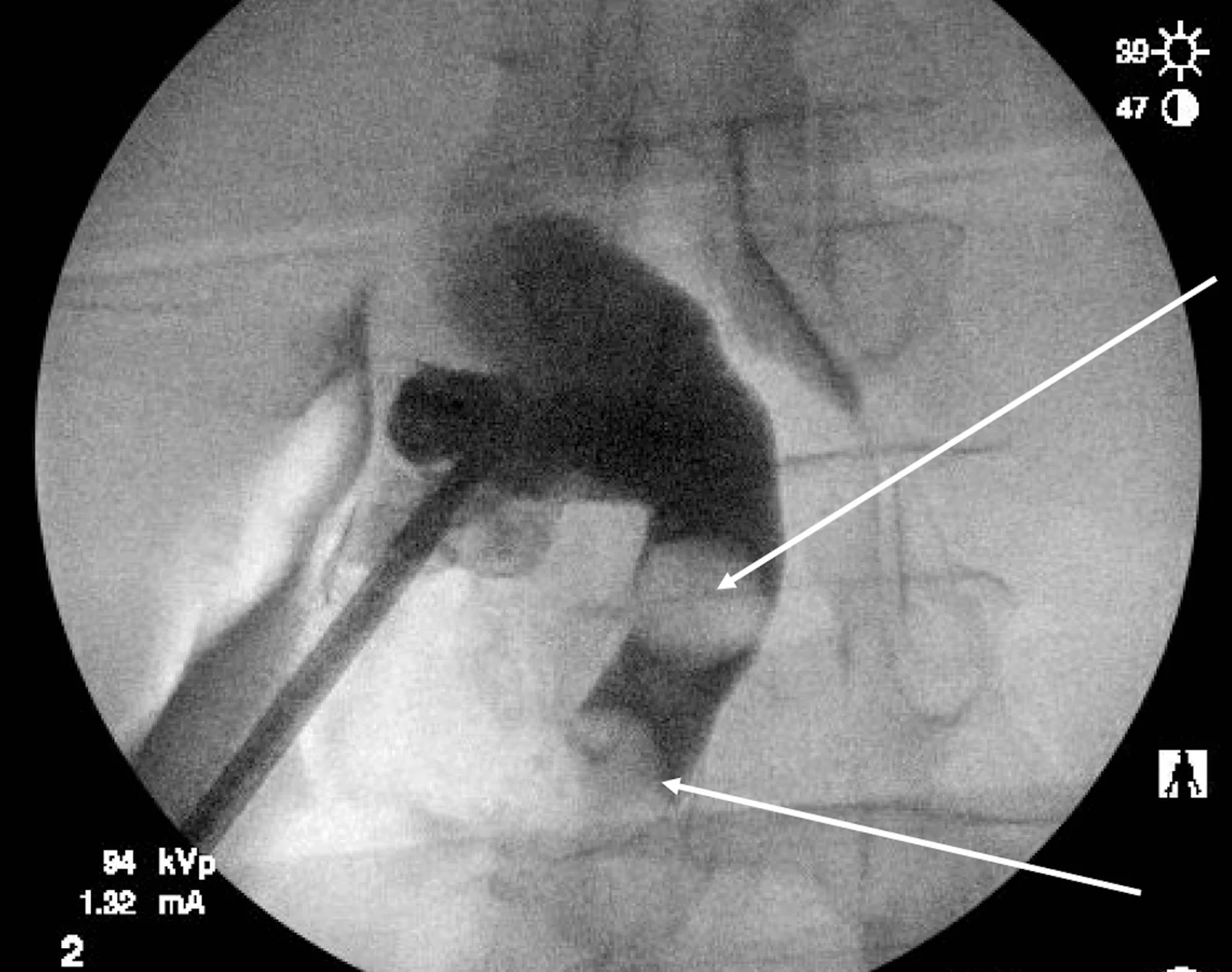
Intraoperative cholangiogram demonstrating two large filling defects indicating common bile duct calculi.

Histopathology results confirmed acute on chronic cholecystitis of the gallbladder and a colonic fistula tract with underlying inflamed granulation tissue and surrounding stromal fibrosis and mucosal ulceration. There was no evidence of abnormal lymph nodes, dysplasia, or malignancy.

Postoperatively, a hepatobiliary iminodiacetic acid (HIDA) scan and then a CT cholangiogram were performed, which revealed a small bile leak (Figure 5), likely from the cystic duct stump, as well as possible remaining calculi in the distal common bile duct (Figure 6). She subsequently underwent endoscopic retrograde cholangiopancreatography (ERCP) and biliary sphincterotomy on postoperative day 8, which achieved complete removal of the choledocholithiasis and placement of a stent in the common bile duct. 

**Figure 5:**
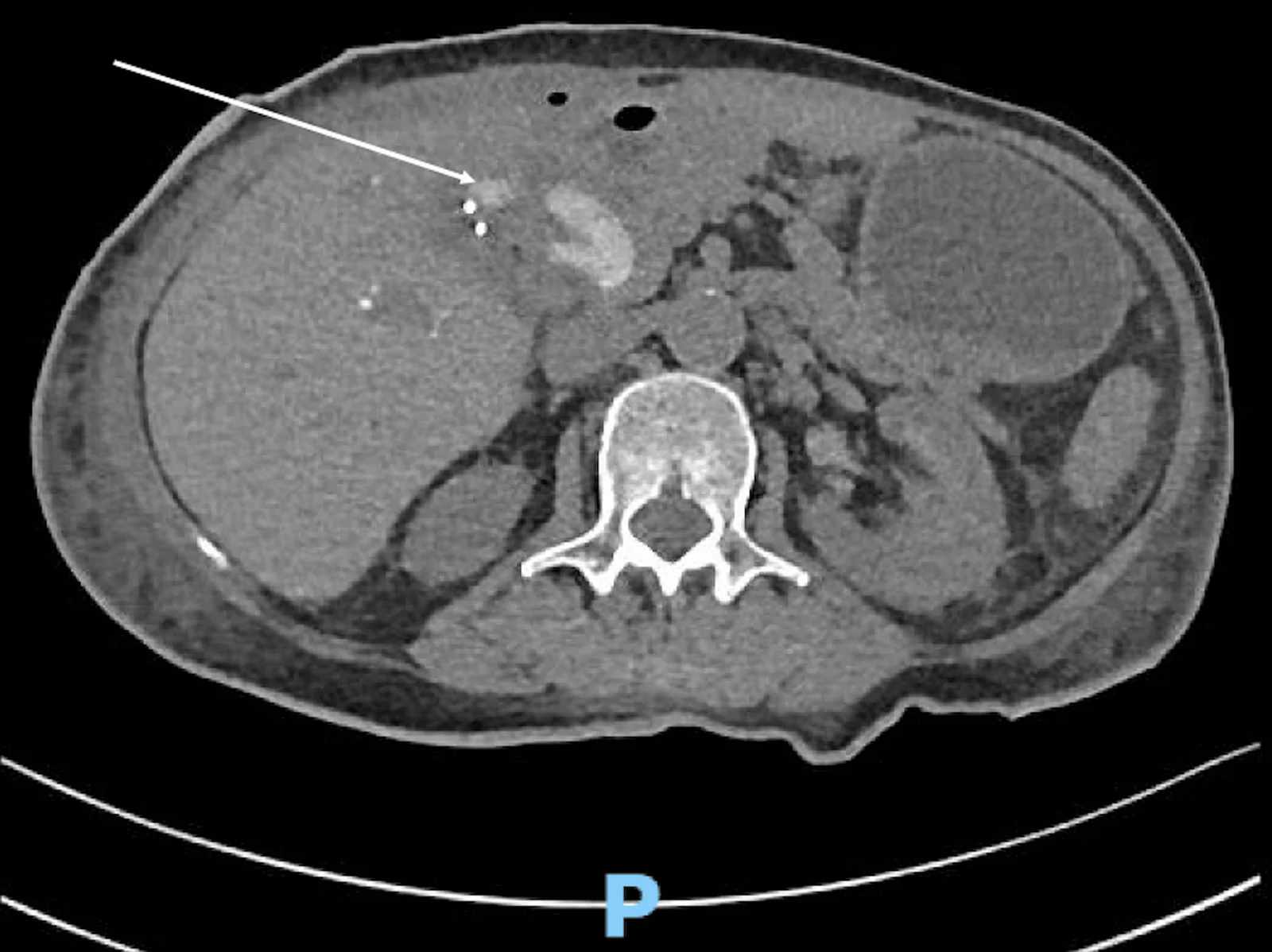
Axial CT of the abdomen and pelvis demonstrating a subtle postoperative bile leak.

**Figure 6:**
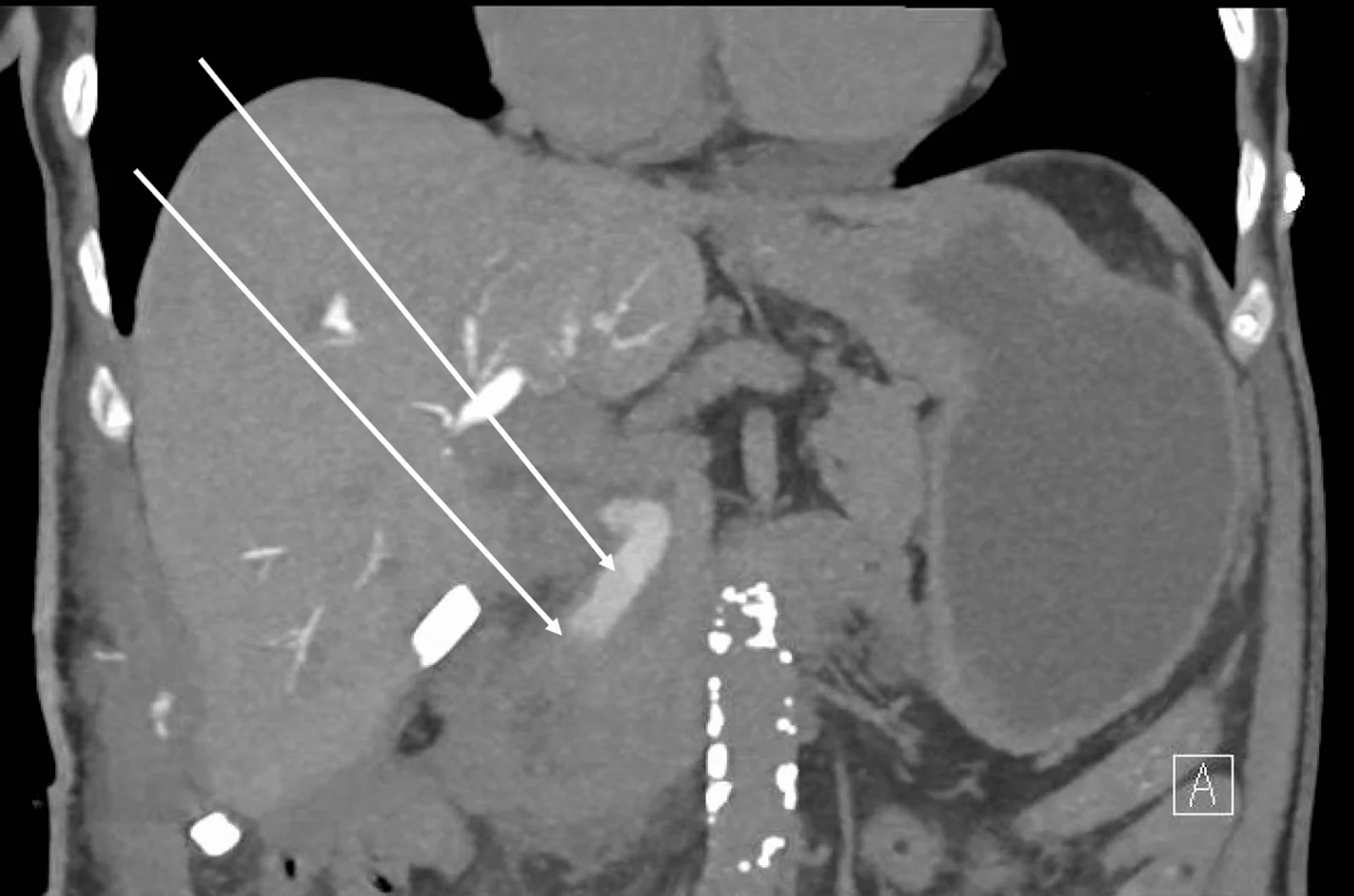
Coronal CT of the abdomen and pelvis demonstrating a distended common bile duct (superior arrow), measuring 14 mm, with poor distal duct opacification (inferior arrow) raising the possibility of a distal duct calculus.

Outcome and follow-up

During her remaining postoperative course, she tolerated an oral diet, was weaned off total parenteral nutrition, and was opening bowels. She required treatment for a urinary tract infection and urinary retention. Her drain tube was removed at one month postoperatively after a successful blue dye test. She remained well and was discharged home with rehabilitation services. Her biliary stent was removed with repeat ERCP at approximately four months postoperatively, with no remaining bile duct calculi. 

## Discussion

A CEF is a rare and abnormal connection between the gallbladder and the gastrointestinal tract [1] and is reported to occur in 3-5% of patients with cholelithiasis [2]. The most common type of CEF is a cholecystoduodenal fistula (71%), followed by CCF (13.8%) and cholecystogastric fistula (7.6%) [3]. CCFs are estimated to occur in approximately one in 1000 cholecystectomies or in 0.06-0.14% of patients undergoing cholecystectomy [4-10]. They are more commonly seen in elderly patients, with a median age at diagnosis of 68.9 years. They have also been reported to be 2.5 times more common in female patients compared to male patients [4].

CCFs usually occur as a complication of chronic cholecystitis, often secondary to gallstones [1,3,11]. The recurrent inflammation leads to gallbladder wall ulceration and ischaemia, with adhesion of the wall to surrounding serosal surfaces. This eventually leads to erosion and subsequent formation of a fistulous connection between the gallbladder and surrounding structures [6-8]. Rarely, CCF can also be secondary to malignancy, with a reported risk of gallbladder malignancy of 15% in CCF cases [5,12]. Other possible aetiologies that have previously been suggested include diverticular disease, inflammatory bowel disease, peptic ulcer disease, trauma, amoebic infections, gastric surgery, previous cholecystostomy, and iatrogenic injury from cholecystectomy [4-7,11,13].

The clinical presentation of CCF is known to be quite vague, which can make diagnosis challenging [4]. Often, they are asymptomatic and only found incidentally during cholecystectomy [1]. Those with symptoms may only report non-specific symptoms such as right upper quadrant pain, nausea/vomiting, dyspepsia, and jaundice [1,7,11]. These may be mistaken for more common differential diagnoses, such as uncomplicated cholelithiasis or cholecystitis, which can delay the diagnosis of CCF.

Chronic diarrhoea is reported to be the most common symptom, with 71% of CCF cases presenting with this [4]. This results from the malabsorption of bile acid which leaks into the colon, disrupting the enterohepatic cycle and resulting in loss of water and electrolytes from the large bowel [5,6,8,11]. Pneumobilia is another finding that may indicate the presence of CCF. Indeed, the triad of chronic watery diarrhoea, vitamin K malabsorption, and pneumobilia is considered to be pathognomonic for CCF diagnosis [5,6,12]. However, studies have not calculated the sensitivity or specificity of this triad, and they are rarely all present [4,10]. Another possible presentation of CCF is recurrent cholangitis due to bacterial overgrowth and inflammation of the gallbladder and biliary tract from the colon [5,11]. Our patient did not present with the triad for CCF, and it was her first known episode of cholangitis.

Rarely, patients may present with complications of CCF. This includes the formation of hepatic abscesses or unexplained per rectal bleeding secondary to gallbladder bleeding [7,13]. CCFs also cause 4.8% of gallstone ileus cases, where a large gallstone enters the gastrointestinal tract, which may also result in a bowel obstruction [1,4]. While the presence of these complications may also raise suspicion of CCF, they were not evident in our patient.

There are multiple diagnostic modalities available, but none have been found to be highly sensitive for CCF, and there have not been studies comparing their diagnostic accuracy [5,10,12]. Most imaging modalities have been involved in the workup of reported CCF cases, including plain X-ray, transabdominal ultrasound, contrast-enhanced CT, MRI or magnetic resonance cholangiopancreatography (MRCP), ERCP, endoscopic ultrasound, biliary scintigraphy, colonoscopy, and barium enema [4-7,10,11]. Generally, imaging findings suggestive of CCF include pneumobilia and a shrunken, atrophic thick-walled gallbladder that is anatomically adjacent to another organ [8,11]. If imaging reveals a possible CCF, one should also consider workup for possible hidden malignancy, such as cross-sectional imaging of the gallbladder or colonoscopy [10]. 

It is worth noting that CCFs are only diagnosed preoperatively in 7.9% of cases [4], and CEF in general is diagnosed preoperatively 43% of the time [7]. This is likely due to their rare occurrence as well as their vague and non-specific clinical presentation. Therefore, they are often found incidentally during cholecystectomy, which may necessitate the conversion of a relatively simple procedure to a more extensive and complex surgery.

The generally accepted management of CCF is surgical, although there is a lack of clear guidelines [1]. A cholecystectomy should be performed as well as excision and closure of the fistula tract, with or without segmental colonic resection [5,10]. Common bile duct exploration may also be performed [9]. Older studies have reported that open surgery was previously the best practice [8], but as advancements have been made in laparoscopic surgical techniques, this has become a safe and effective alternative, with quicker recovery, decreased length of stay, and decreased morbidity [1]. Some authors have raised concern about the possible increased operative time for laparoscopic surgery and the increased risk of needing to convert to open [6,8]. However, there is a wide discrepancy in conversion rates reported in the literature, ranging between 6.3% and 60% [7]. Ng et al. [3] conducted a retrospective study of 145 CEF patients and found a conversion to open rate of 22.1%. More recently, robotic surgery has also emerged as a potential surgical alternative [9]. The choice of surgical intervention (laparoscopic versus open versus robotic) would depend on the patient’s clinical condition, surgeon experience, and the available resources.

If CCF is left untreated, complications may arise, including acute cholangitis, biliary peritonitis, and biliary cirrhosis, which carry an overall mortality rate of 10-15% [4,6,10]. While there have been a few authors who have previously suggested that CCF can be managed with supportive care and symptomatic management [11], the risk of these complications supports the decision to proceed to surgery if CCF is identified, in order to minimise the risk of sepsis from bacterial translocation [9,13,14]. In addition, if CCF is missed intraoperatively, this can lead to complications such as colonic perforation, faecal peritonitis, sepsis, or death [7,9]. 

## Conclusions

We present a case of a CCF, which was diagnosed preoperatively in the setting of workup for acute cholangitis and underwent successful surgical management. The rare occurrence of this condition highlights the importance of having a high index of clinical suspicion preoperatively as well as maintaining surgical flexibility if it is encountered during an operation. Early recognition and intervention of CCF are important to prevent life-threatening complications.
